# Exposure of α-Synuclein Aggregates to Organotypic Slice Cultures Recapitulates Key Molecular Features of Parkinson's Disease

**DOI:** 10.3389/fneur.2022.826102

**Published:** 2022-02-16

**Authors:** Serge Moudio, Fredrik Rodin, Nazira Jamal Albargothy, Urban Karlsson, Juan F. Reyes, Martin Hallbeck

**Affiliations:** ^1^Department of Clinical Pathology and Department of Biomedical and Clinical Sciences, Linköping University, Linköping, Sweden; ^2^Department of Biomedical and Clinical Sciences, Linköping University, Linköping, Sweden

**Keywords:** Parkinson's disease, α-synuclein, PFF, organotypic slice cultures, autophagy, 3R, Lewy bodies, model of CNS disease

## Abstract

The accumulation of proteinaceous deposits comprised largely of the α-synuclein protein is one of the main hallmarks of Parkinson's disease (PD) and related synucleinopathies. Their progressive development coincides with site-specific phosphorylation, oxidative stress and eventually, compromised neuronal function. However, modeling protein aggregate formation in animal or *in vitro* models has proven notably difficult. Here, we took advantage of a preclinical organotypic brain slice culture model to study α-synuclein aggregate formation *ex vivo*. We monitored the progressive and gradual changes induced by α-synuclein such as cellular toxicity, autophagy activation, mitochondrial dysfunction, cellular death as well as α-synuclein modification including site-specific phosphorylation. Our results demonstrate that organotypic brain slice cultures can be cultured for long periods of time and when cultured in the presence of aggregated α-synuclein, the molecular features of PD are recapitulated. Taken together, this *ex vivo* model allows for detailed modeling of the molecular features of PD, thus enabling studies on the cumulative effects of α-synuclein in a complex environment. This provides a platform to screen potential disease-modifying therapeutic candidates aimed at impeding α-synuclein aggregation and/or cellular transmission. Moreover, this model provides a robust replacement for *in vivo* studies that do not include behavioral experiments, thus providing a way to reduce the number of animals used in an accelerated timescale.

## Introduction

Parkinson's disease (PD) is the second-most prevalent neurodegenerative disorder that affects ~2 to 3% of individuals over the age of 65. The main neuropathological hallmarks of this disease are the formation of Lewy bodies (LBs) and Lewy neurites (LNs), intracellular inclusions consisting mainly of α-synuclein aggregates, which are predominantly located in the substantia nigra pars compacta (SNpc) and other vulnerable brain regions (1–3). Subsequent neuronal loss and dopamine deficiency cause progressive motor deficits observed in PD patients (4, 5). Inherited forms of PD are the result of missense mutations within the α-synuclein gene (*SNCA*) which lead to expression of aberrant α-synuclein proteins (A30P, E46K, G51D, A53T/E) (6), highly resistant to classical degradation pathways within the cell (7). It is believed that the subsequent accumulation of α-synuclein in vulnerable brain regions may be responsible for PD pathogenesis (8). However, the cause of these detrimental events likely involves dysfunction of multiple cellular pathways responsible for proteostasis, cellular trafficking, and the proper function of mitochondria, axons, and synapses (2, 8).

The α-synuclein protein is a small molecule of 140 amino acids primarily located at the presynaptic terminals where it is thought to be involved in neurotransmitter release (9). In pathological conditions, misfolded α-synuclein has been demonstrated to aggregate, induce self-seeding and promote the propagation of protein aggregates from cell-to-cell (10, 11), suggesting that efforts aimed at removing misfolded α-synuclein or reducing cellular transmission could have important therapeutic applications. Thus, a better understanding of the pathological disease mechanisms that lead to disease onset could accelerate drug development for PD. In this study, we explored the potential of using organotypic slices as a model to investigate the cellular and molecular mechanisms of PD in a multicellular system. Organotypic slice cultures prepared from explanted brain regions represent a physiologically relevant experimental three-dimensional model of the central nervous system (CNS) and are increasingly used in molecular biology, electrophysiology, and immuno-histochemical studies (12–14). Excitingly, several studies have explored the used of organotypic slices as a model for different CNS diseases including PD (15–18). For instance, viral expression of human wild type or mutant α-synuclein harboring genetic mutations that lead to early PD onset was shown to promote α-synuclein inclusion formation modeling those identified in human PD brains (19, 20). Daviaud and colleagues lesioned the medial forebrain bundle in organotypic slices to model dopaminergic degeneration over time (19). By co-culturing astrocytes with organotypic slices, Loria and colleagues demonstrated the transfer of α-syn from neurons to astrocytes and the degradation capacity of astrocytes for α-synuclein aggregates (21). Shrivastava et al. characterized the inter-neuronal spreading of different α-syn fibrillar strains (22) while Roux et al. demonstrated that endogenous α-syn expression is necessary and sufficient for α-syn seeding and spreading (23). Taking advantage of the highly complex synaptic connectivity, neuronal function and circuitry provided by hippocampal slices, Elfarash et al. successfully modeled α-synuclein aggregation and inter-neuronal spreading initiated by direct microinjection of α-synuclein pre-formed filaments (PFFs) days after α-synuclein treatment (24). Similarly, in another elegant study, Wu et al. further demonstrated that neuronal activity modulates α-synuclein aggregation and spreading days after α-synuclein treatment. Interestingly, they also demonstrated that α-synuclein assembly uptake was higher within the CA3 brain region compared to CA1 (25). Recently, Barth et al. (26), also demonstrated that the seeding properties of α-synuclein following injection can be studied more long-term both in murine and human brain slice cultures.

In the current study, we cultured hippocampal organotypic slice cultures for long time periods in the presence of different α-synuclein aggregation states, either separately [monomers or fibrils (PFF)] or in combination (monomer/PFF), in levels that do not cause acute toxicity. This experimental model recapitulated the typical cellular features of PD pathology that include α-synuclein aggregation, cellular toxicity, mitochondrial damage, and cellular death. Particularly, this included site-specific phosphorylation at serine-129 (pS129) as seen in PD brains. Taken together, we describe a simple and reliable method for the preparation of organotypic hippocampal slice cultures to investigate how the addition of α-synuclein in levels that do not cause acute toxicity impact cell viability over long periods of time, enabling the study of the cumulative effects of α-synuclein in a complex environment. This will be useful to further advance our understanding of the interplay between α-synuclein accumulation and toxicity to evaluate potential therapeutic interventions.

## Materials and Methods

### Animals

All experimental procedures were carried out in compliance with the regional ethics committee for animal research (ID 1276) and followed EU directive (2010/63/EU), with specific efforts made to minimize suffering and stress. Pregnant female Sprague–Dawley rats were obtained from Janvier-Labs (France) and housed in standard transparent plastic cages with free access to food and water with a 12-h light/dark cycle. Temperatures were maintained between 24–26°C (±2) and humidity levels at 55% (±5). Behavioral enrichment including nesting materials, cardboard tubes, and soft wood to gnaw were provided. Both male and female pups were used in this study.

### Organotypic Slice Culture Preparation

Organotypic hippocampal slice cultures were prepared from Sprague-Dawley rats at postnatal days 6–8 according to the interface culture method (12). Following decapitation, brains were removed and placed in ice-cold Gey's balanced salt solution (GBSS, Sigma-Aldrich, Sweden), supplemented with 25 mM D-glucose (Scharlab, Spain) and 1 mM Kynurenic acid (KYN, Sigma-Aldrich). The pH was adjusted to 7.4 and the GBSS solution was kept cool under sterile conditions. The brain was divided into two hemispheres by a single cut along the interhemispheric fissure and the cerebellum, brainstem and a segment of the frontal cortex were discarded. The hippocampus, together with the entorhinal and perirhinal cortex were immersed in cold GBSS solution and 400 μm sagittal slices were produced using a Vibratome (Leica, Sweden). Three consecutive slices were placed onto 0.4 μm hydrophilic Millicell culture inserts (Millipore, Germany) in 6-well culture plates (Corning, Germany). Each well contained 1 ml of sterile pre-warmed culture medium composed of MEM (50%), HBSS (1%), HEPES buffer solution (1%), amphotericin B (1%), glutamax supplement (0.5%) (all from Gibco, Germany), L-glutamine (1%), penicillin/streptomycin (1%) (Lonza, Sweden), 25 mM D-glucose (1.5%) and heat-inactivated bovine serum (25%) (GE Healthcare Life Science) in ultrapure Milli-Q H_2_O (18%). The pH was then adjusted to 7.4 and the solution was filter sterilized. Prior to use, culture medium was equilibrated at 95% O_2_, 5% CO_2_ and 37°C for a minimum of 3 h. The day following dissection, inserts were transferred to new 6-well culture plates containing 1 mL of pre-warmed fresh sterile culture media. Hippocampal slices cultures were subsequently maintained for 2 weeks prior to treatment and media was replaced every 48 h. Subsequent experiments were performed on 49 different hippocampal slice culture preparations, each yielding 18 to 36 slices.

### α-Synuclein Treatment of Organotypic Slices

At 14 days *in vitro* (DIV), 3 days prior to the beginning of the treatment and to avoid serum interference, inserts were placed in filter-sterilized serum-free media (pH 7.4) made up of DMEM/F12 (69%), B27 supplement (0.5%) (Gibco), HBSS (25%), HEPES (1%), L-glutamine (1%), 25 mM D-glucose (1.5%), Amphotericin B (1%), penicillin/streptomycin (1%). Prior to use, the serum free media was equilibrated at 95% O_2_, 5% CO_2_, and 37°C for a minimum of 3 h. Every 48 h, inserts were transferred to new 6-well culture plates containing 1 mL of fresh sterile serum free medium, and the supernatant was collected and stored at −20°C for further analysis. At every media change, slices were cultured in a combination of 2.5 μM α-synuclein monomers (human recombinant, Alexotech, Sweden) and/or 500 nM α-synuclein pre-formed fibrils (PFF) to induce α-synuclein pathology while control slices only received fresh media. To generate pre-formed fibrils, we first labeled WT proteins with ATTO as we previously reported (27). Briefly, we incubated ATTO-labeled monomers at a concentration of 4.0 mg/mL at 37°C for 5 days in an Eppendorf SS mini-shaker (Eppendorf, Germany) with constant shaking at 1,000 RPM, followed by sonication and further validation using Thioflavin T as previously reported (14, 27).

### Electrophysiological Recordings

To confirm the functional electrical properties of organotypic slices cultured for 57 DIV, we recorded spontaneous synaptic currents and evoked action potentials activity. Recordings of pyramidal neurons in cell layers of CA1 or CA3 were done in whole-cell current-clamp and voltage-clamp mode. At the end of the experimental period, culture insert membranes were cut out to allow transfer of slices to a submerged recording chamber continuously super fused with pre-warmed (34°C) artificial cerebrospinal fluid (aCSF) containing (in mM): NaCl 127, KCl 1.8, CaCl_2_ 2.4, MgSO_4_ 1.3, KH_2_PO_4_ 1.2, NaHCO_3_ 26, glucose 15, bubbled with 95% O_2_ and 5% CO_2_. Slices were secured with a harp slice grid (ALA Scientific Instruments, Scientifica, United Kingdom). Patch pipettes were of borosilicate glass and had a resistance of 4–6 MΩ. The intracellular solution contained (in mM): 120 K-gluconate, 10 KCl, 1 EGTA, 10 HEPES, 4 Mg-ATP, 0.3 Na-GTP. pH was set to 7.3 using KOH.

### Quantification of Extracellular Toxicity

Cellular toxicity in organotypic hippocampal slice cultures following α-synuclein treatment was assessed by measuring the extracellular lactate dehydrogenase released (LDH) in culture media using an LDH assay kit (Thermo Fisher Scientific, Germany). Following manufacturer's instructions, the enzymatic reaction was measured using a multimode microplate reader (Sparks 10, Tecan, Switzerland) in a 96-well plate. To determine LDH activity, the absorbance at 680 nm (background signal) was subtracted from the absorbance at 490 nm for each value. Data were then normalized to the positive control conditions for each sample and for each day and expressed as a percentage of total LDH activity. To minimize experimental bias, all LDH assays were performed blinded, and the treatment conditions were revealed only after the statistical analysis were complete.

### Proteostat Aggregate Detection Assay

The PROTEOSTAT® Protein aggregation assay (Enzo Life Sciences, Germany) was used to measure α-synuclein aggregates in solution as described by the manufacturer's instructions. Briefly, hippocampal slices supernatant was used to detected protein aggregates using 0.5 μl of reagent added to 1 ml of assay buffer provided with the kit and added to supernatant solutions concentrated through ultrafiltration (0.22 μm Millipak filters, Millipore) and collected from the different culture conditions after 35 DIV. The luminescence signals were then measured using a plate reader (Spark 10, Tecan).

### Quantification of Mitochondrial Toxicity

Assessment of mitochondrial toxicity was performed using the Mitochondrial ToxGlo Assay to quantify cellular ATP levels according to the manufacturers' instructions (Promega, Germany). Briefly, for each condition a cell suspension containing 200,000 cells per ml were prepared from chopped tissue sections and supernatant. Fifty microliter cell suspension were incubated with 20 μl of cytotoxic reagent in 96-well plates for 30 min at 37°C. Subsequently, 100 μl ATPase reagent was added and plates were shaken for 1 min, before measuring the luminescence signal using a plate reader (Spark 10, Tecan).

### Cellular Death Quantification

Cell death in the tissue was assessed by monitoring the cellular uptake of the nuclear marker propidium iodide (PI, Sigma-Aldrich). PI imaging was carried out at the end of the experimental period and slices were incubated with 1 μM sterile PI diluted in serum-free culture medium for 3 h before imaging. Cellular uptake of PI was recorded by fluorescence microscopy on a DMi8 wide field fluorescence microscope using a rhodamine filter (Leica, Germany). All fluorescence images were acquired with identical settings and lighting using a 10 × air objective (0.3 numerical aperture). For assessment of PI uptake, fluorescence intensity was quantified using the software ImageJ 1.52.e (National Institute of Health, USA) and expressed in arbitrary units (A.U.). Following baseline measurements, slices where further incubated with the glutamate agonist kainic acid (100 μM, Abcam, United Kingdom) for 24 h to induce cell death, and imaged again to establish the maximum amount of cell death quantifiable. For each slice, levels of fluorescence were then expressed as a percentage of maximum cell death.

### Immunohistochemistry

Hippocampal slices were either cryo-protected with a 15–30% sucrose gradient in PBS over 2 days or fixed and paraffin-embedded to preserve tissue morphology. For cryo-sectioning, slices were embedded in OCT embedding matrix (Thermo Fisher Scientific), frozen on dry ice, and stored at −80°C. Sections were cut at −20°C with a thickness of 15 μm using a cryostat (CM3050S, Leica), collected onto 1 mm microscope slides and stored at −20°C until further use. For paraffin embedding, slices were fixed in 4% PFA solution overnight at room temperature, then processed in up-gradient series of alcohol, xylene and infiltrated in paraffin using a benchtop Tissue Processor Leica TP1020 (Leica). Paraffin-embedded slices were then sectioned with an automated Rotary Microtome HM355S (Thermo Fisher Scientific, Germany) and antigen retrieval was performed for 20 min at 97°C in target retrieval buffer using a Dako PT Link (Agilent, Sweden). For immunohistochemistry, slices were incubated with 3% H_2_O_2_ (Sigma-Aldrich) for 15 min, washed with PBS, blocked for 30 min at room temperature (5% BSA in 0.025% triton X-100; Sigma-Aldrich), and subsequently incubated with primary antibody (mouse pS129, 1:2,000, Wako, Japan: 015-25191) in 0.025% triton X-100 in PBS at 4°C, overnight in a moist chamber. The next day, slices were washed and incubated with biotinylated anti-mouse IgG1 secondary antibody (1:400, Vector Laboratories, United Kingdom) for 1 h at room temperature. Subsequently, slices were washed in PBS then incubated in ABC reagent (1:200 in 0.025% PBS-triton X-100; Vectastain Kit, Vector Laboratories) for 1 h at room temperature. Following PBS washes, slices were treated with 3, 3-diaminobenzidine (DAB) HRP substrate mixed with Urea Hydrogen Peroxide tablets (Sigma-Aldrich) in 1 mL MilliQ H_2_O, then rinsed with 0.1 M sodium acetate in distilled H_2_O (pH 6.0), followed by PBS washes. Slices were then dehydrated through a series of alcohols (50, 70, 95, and 100%) for 1 min each, followed by xylene for 5 min and mounted using DPX (Sigma-Aldrich), with coverslips sealed using nail polish and microscopic slides were kept at 4°C pending imaging.

### Immunofluorescence

To visualize the presence of specific proteins within the organotypic hippocampal slices, preparation for immunostaining was carried out as described previously (16, 17). At the end of the experimental period, tissue sections were fixed in 4% PFA solution for 15 min at room temperature followed by 20% methanol/PBS solution for an additional 5 min. Slices were then permeabilized with 0.5% triton X-100 in PBS overnight at 4°C. The following day, sections were rinsed in PBS and blocked using a 20% BSA/PBS solution for 4 h at room temperature, followed by incubation with primary antibody in a 5% BSA/PBS solution overnight at 4°C. The primary antibodies used for immunostaining include; rabbit monoclonal anti-α-synuclein (1:1,000, Invitrogen: 701085), rabbit polyclonal anti-LC3 II (1:1,000, Novus Biologicals, Germany: NB600-1384), rabbit polyclonal anti-GFAP (1:1,000, Abcam: ab7260), rat monoclonal anti-CD11b (1:1,000, Abcam: ab8878), chicken polyclonal anti-MBP (1:1,000, Abcam: ab123499), mouse anti-pS129 (1:1,000, Wako: 015-25191), and guinea pig polyclonal anti-p62 (SQSTM1) (1:1,000, Nordic Biolabs, Sweden: 318-GP62-C). The next day, sections were rinsed with PBS and incubated with appropriate Alexa Fluor conjugated secondary antibodies (1:1,000) in a 5% BSA/PBS solution for 4 h at room temperature, or overnight at 4°C. Following PBS washes, tissue sections were stained with DAPI solution (1 mg/mL, Invitrogen: D1306), staining DNA of the cell nucleus or DRAQ7 (5 μM, Abcam: ab109202), staining the nuclei in dead and permeabilized cells, at room temperature for 15 min rinsed then mounted on microscopic slides using ProLong Gold antifade reagent (Invitrogen). To provide reference levels for maximal levels of LC3-II and p62 (positive controls), organotypic hippocampal slices were treated with 200 nM rapamycin (Sigma-Aldrich: R8781) dissolved in dimethylsulfoxide (DMSO). After immunostaining the fluorescence levels of LC3II and p62 were analyzed, respectively, and used as maximum values compared to the values obtained from each treatment.

### Image Acquisition and Processing

Image acquisition was performed using a Zeiss LSM 800 Airyscan confocal laser-scanning microscope (Inverted) with ZEN 2.3 software (Carl Zeiss, Oberkochen, Germany) equipped with solid-state diode lasers (excitation wavelengths 405, 488, 561, and 640 nm) for DAPI, FITC, Rhodamine and Cy5-like fluorophores. Channel mode visualization was completed using a 40 × objective (1.4 numerical aperture) or 63 × objective (1.4 numerical aperture), both oil immersion objectives. For protein quantification, fluorescence images were acquired on an inverted wide field Leica DMi8 (Leica) with a metal halide excitation source, using a 10 × air objective (0.3 numerical aperture) and a Leica DFC 3000 G CCD camera, always using identical settings in the LASX software (Leica).

### Western Blot Analysis

For analysis of supernatants, cryotubes were thawed at room temperature before pelleted at 2,000 rpm for 2 min to remove debris. Pellets were then lysed in ice-cold buffer (50 mM Tris-HCl, 150 mM NaCl, 1% Triton-X 100, 0.5% sodium deoxycholate, and 0.1% SDS, supplemented with phosphatase (PhosStop; Roche, Switzerland) and protease inhibitors (Halt; Thermo Fisher Scientific), followed by a brief sonication. Lysates were then centrifuged at 10,000 rpm at 4°C for 10 min and supernatants were collected and quantified using the Bio-Rad DC Protein Assay Kit (Bio-Rad, Sweden). Samples (20 μg total protein) were mixed with DTT (final concentration 0.1 M, Sigma-Aldrich) and boiled at 95°C for 5 min then loaded onto a 4–20% SDS Clear-Page gel (CBS Scientific, USA). Proteins were then transferred to nitrocellulose membranes (iBlot; Invitrogen, Sweden) then blocked with 5% milk (BioRad) in Tris-buffered saline with Tween 20 (0.1%, Sigma-Aldrich). Membranes were developed with primary antibodies [rabbit monoclonal anti-α-synuclein (1:10,000, Invitrogen: 701085), anti-ps129 (1:10,000, Wako: 015-25191), anti-β-actin (1:10,000, Sigma-Aldrich: AC-15)] and appropriate horseradish peroxidase (HRP) conjugated secondary antibodies (Dako, Sigma-Aldrich). Immunoreactions were visualized with Clarity ECL (BioRad) on a ChemiDoc MP imaging system (BioRad) and the integrated intensity of each band was calculated using computer-assisted densitometry analysis using ImageJ using β-actin as the loading control.

### Transmission Electron Microscopy

Tissue sections were fixed with 2% glutaraldehyde in 0.1 M sodium cacodylate buffer (pH 7.4, Sigma-Aldrich), for 2 h at room temperature. Samples were then washed with 0.1 M sodium cacodylate buffer and post-fixed for 1 h using 2% osmium tetroxide (Thermo Fisher Scientific). To enhance contrast, en bloc staining with 2% uranyl acetate (Agar Scientific, United Kingdom) in 50% ethanol was performed prior to dehydrating the samples in a series of ascending concentrations of ethanol and acetone. Following a two-step infiltration, tissues sections were an embedded using the Spurr Low Viscosity Embedding Kit (Sigma-Aldrich). Blocks were initially trimmed and sectioned using a UC7 ultra microtome (Leica, Germany), ultrathin sections (60 nm thick) were then collected onto formvar-coated copper slot grids (Agar Scientific) and counter-stained with uranyl acetate and lead citrate. Images were taken using a 100 kV (JEM 1230, JEOL Ltd., Tokyo, Japan).

### Statistical Analysis

All statistical analyses and result figures were produced using GraphPad Prism software 8.0, with data representing the mean ± standard error of the mean (SEM), taken from independent cultures. Unless specified, comparisons between treatment conditions were made using 1 or 2-way ANOVA with either Tukey's or Bonferroni's multiple comparison *post-hoc* analysis. The number of independent cultures or slices (*n*) used are listed in each figure legend. Values of *p* < 0.05 were considered to be statistically significant.

## Results

### Cellular Structure and Viability Are Preserved in Cultured Organotypic Slices Over Time

**Figure 1:**
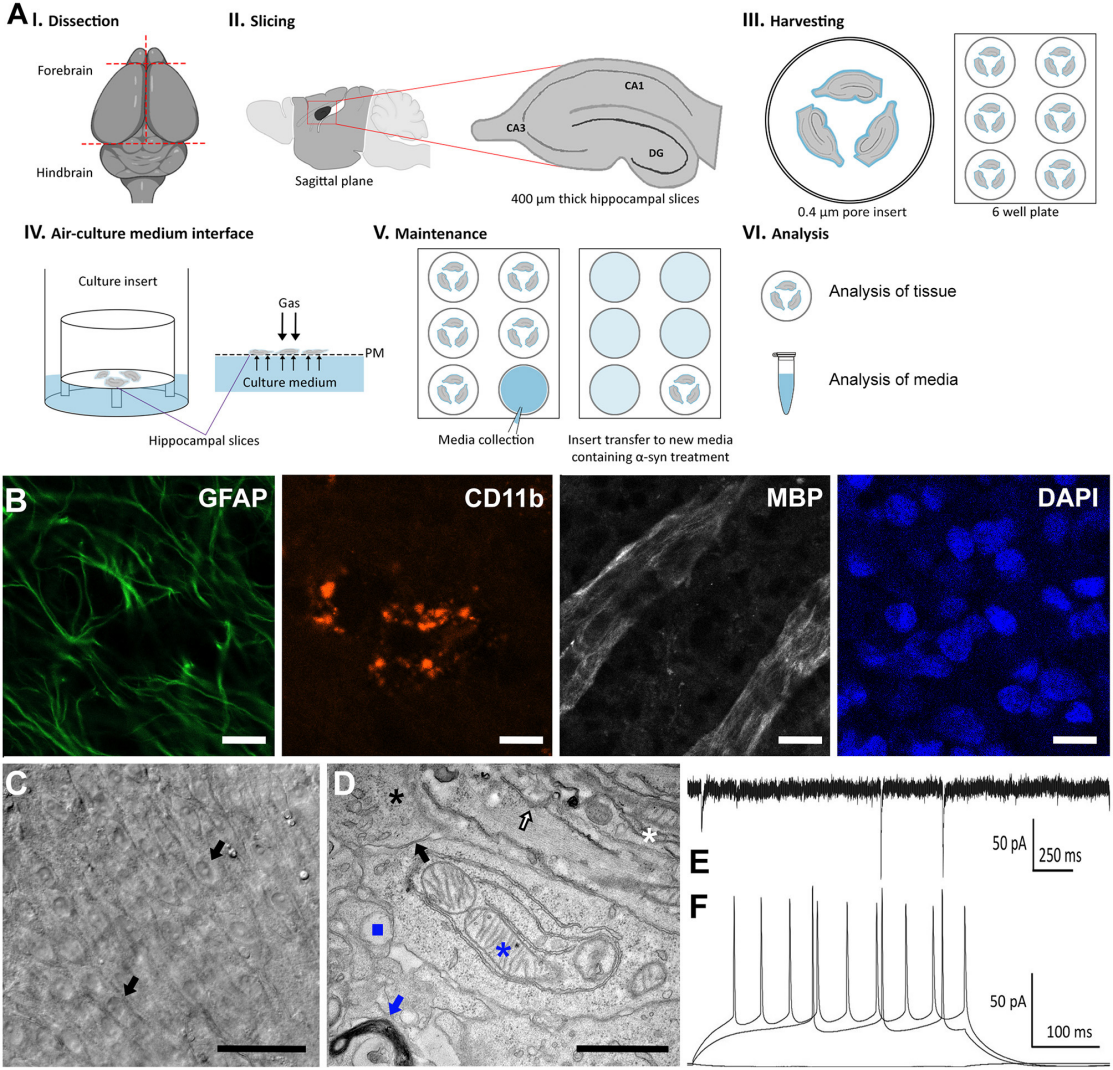
Characterization of an organotypic slice model to study PD *ex vivo*. **(A)** Schematic diagram depicting the preparation, maintenance, and analysis for organotypic slice cultures to model PD. Hippocampal tissue were isolated from postnatal days 6–8 Sprague-Dawley rat brains (I). Four-hundred micrometer sagittal slices were produced using a Vibratome (II) and placed onto 0.4 μm hydrophilic culture inserts in 6-well plates containing 1 ml of pre-warmed media (III), cultured in an atmosphere composed of 95% O_2_, 5% CO_2_ at 37°C (IV) with 3 media changes per week. After 2 weeks, the initial serum media was replaced with serum free media containing α-synuclein, which was collected at every media change (V). At the end of the experimental period, analysis was conducted on media collected throughout the experiment and on the tissue (VI). **(B)** Representative confocal immunofluorescence (40x) images of organotypic slice cultures immunolabeled with the astrocyte marker, glial fibrillary acidic protein (GFAP, green), the microglia marker, cluster of differentiation molecule 11B (CD11B, red) and myelin basic protein (MBP, white) and counterstained with DAPI (blue). The presence of labeling varies through the tissue and thus optimal depth levels through the z-stack were selected to illustrate the presence of each marker. Scale bar = 20 μm. **(C)** Representative wide-field images of organotypic slices following 57 DIV. Picture is showing different layers of cells, including pyramidal cells (black arrows). Scale bar = 50 μm. **(D)** Representative TEM micrograph of organotypic slice cultures showing nuclei (black asterisk), intact synapses (black arrow), myelinated bodies (white asterisk), mitochondria (blue asterisk), lysosomes (blue square), endoplasmic reticulum (white arrow), and golgi system (blue arrow), scale bar = 1 μm. **(E)** Representative whole patch clamp recording from the pyramidal cell layer of the CA3 region at 57 DIV. Spontaneous synaptic currents showing functional synapses. Holding membrane potential −70 mV. **(F)** Train of actions potentials evoked by current injections (200 and 280 pA positive steps) showing that the neuron is excitable with resting membrane potential of −80 mV.

In this study, we characterized a method for culturing organotypic hippocampal slices from Sprague-Dawley rats at postnatal days 6–8 to study human α-synuclein accumulation and toxicity over long time periods. Given that α-synuclein accumulation and spreading within the mouse brain is observed approximately within a month post-α-synuclein injection (28), we assessed whether we could culture organotypic hippocampal slices for extended periods of time to visualize α-synuclein aggregation and transmission. As shown in the schematic illustration (Figure 1A, I-VI), rat brains were isolated under sterile conditions and immediately placed onto hydrophilic culture inserts in 6-well plates containing 1-ml of pre-warmed media (37°C).

Following an initial 2-week period in hydrophilic culture inserts containing media supplemented with serum, we then tested whether slices could be cultivated in the absence of serum as previously done for cellular models during cell differentiation (29, 30). We cultured slices with or without serum starting from 14 days *in vitro* (DIV) for up to eight additional weeks and observed no significant difference in tissue structure or viability in the absence or presence of serum (data not shown). Based on this observation, we decided to culture hippocampal slices in serum free media starting from DIV14 to prevent serum interference in our assays specifically with regards to cellular death (31) and/or pharmacodynamics (32) where such interferences have been reported. Following protocol optimization, we determined that three full media changes per week in fresh 6-well plates provided the most optimal conditions. The conditioned media was collected after each media change.

We next characterized our serum-free hippocampal slice model system using immunohistochemical analysis and as expected, we observed normal cellular morphology with typical complex architecture for up to 57 DIV as previously reported for cultures containing serum (33). Indeed, immunofluorescence analysis revealed a robust expression of cellular markers including the glial fibrillary acidic protein (GFAP), cluster of differentiation molecule 11b (CD11b), and the myelin basic protein (MBP), conventional glial markers for astrocytes, microglia, and oligodendrocytes, respectively (Figure 1B). Brightfield microscopy further revealed the cell bodies of pyramidal neurons within the CA1 and CA3 regions on the tissue surface as described previously for similar explants cultivated in the presence of serum (Figure 1C) (34). Moreover, high magnification electron micrographs demonstrated a very dense field of cellular matter such as nuclei (black asterisk), intact synapses (black arrow), and myelinated bodies (white asterisk). Upon closer examination, evidence of viable mitochondria (blue asterisk), lysosomes (blue square), Golgi (blue arrow), and endoplasmic reticulum (white arrow), as well as various vesicular structures were visible (Figure 1D).

Furthermore, electrophysiological recordings revealed spontaneous synaptic currents showing functional synapses (Figure 1E). Finally, we observed a train of action potentials evoked by current injections (200 and 280 pA positive steps) further demonstrating that neurons within the CA1 and CA3 regions are excitable with a resting membrane potential of −80 mV (Figure 1F). Taken together, characterization of our serum-free hippocampal slice model confirmed that slices cultured under serum free conditions are viable, display well-preserved physical architecture (Figures 1B–D), normal electrophysiology responses (Figures 1E,F), limited toxicity and cellular death under control conditions.

### The Exogenous α-Synuclein Organotypic Slice Model Recapitulates key Molecular Features of PD

**Figure 2:**
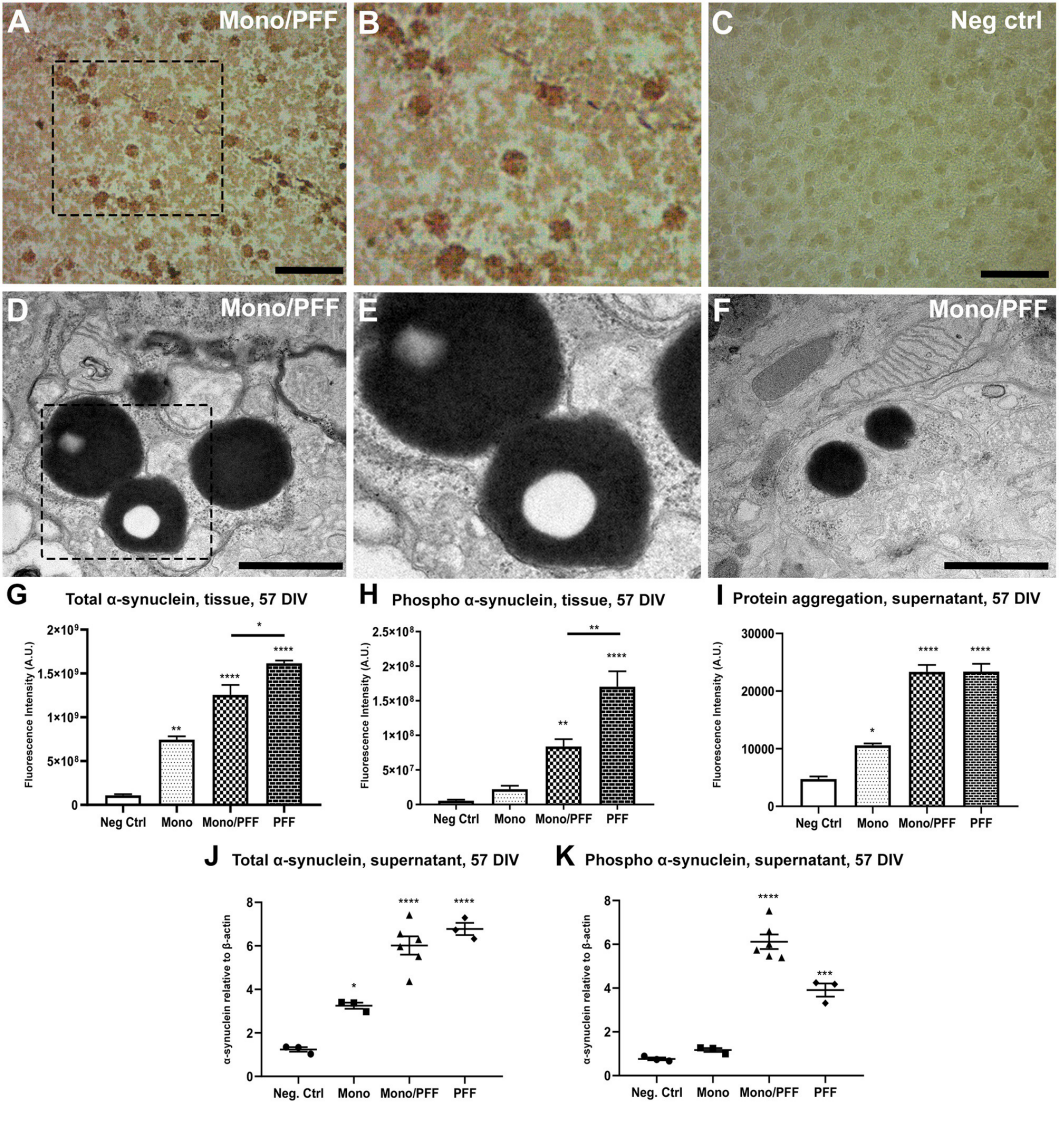
Exogenous α-synuclein is taken up by organotypic slices, causing accumulation, aggregation and phosphorylation. **(A)** Representative immunohistochemical staining of organotypic slice cultures treated with monomeric/PFF α-synuclein showing the presence of pS129 (phosphorylated α-synuclein) positive neurons and neurites following 57 DIV. Scale bar = 50 μm. **(B)** 2 × Magnified image of marked area in **(A)**. **(C)** Untreated negative control with no identified pS129 labeled structures organotypic slice cultures following 57 DIV. Scale bar = 50 μm. **(D)** Representative TEM of organotypic slice cultures treated with monomeric/PFF α-synuclein and following 57 DIV. Note the LB-like electron dense inclusion bodies in organotypic slice cultures. Scale bar = 1 μm. **(E)** Magnification from marked area in **(D)**, note the amorphous granular composition with autophagosomes double membrane. **(F)** Representative TEM of Lewy-bodies like electron-dense inclusion bodies in organotypic slice cultures treated with monomeric/PFF α-synuclein, following 57 DIV. Scale bar = 1 μm. **(G)** Semi-quantitative analysis of α-synuclein immunofluorescence in organotypic slice cultures following 57 DIV (*n* ≥ 8). **(H)** Semi-quantitative analysis of phosphorylated α-synuclein immunofluorescence organotypic slice cultures following 57 DIV (*n* ≥ 7). **(I)** Levels of total aggregated protein were determined within the supernatant of organotypic slice cultures following 57 DIV by fluorometric Proteostat® assay (*n* ≥ 4). **(J)** Western blot analysis of α-synuclein within the supernatant of organotypic slice cultures following 57 DIV (*n* ≥ 8). **(K)** Western blot analysis of phosphorylated α-synuclein within the supernatant of organotypic slice cultures following 57 DIV (*n* ≥ 7). Values represent ± S.E.M. of at least three independent experiments. *p < 0.05, **p < 0.01, ***p < 0.001, ****p < 0.0001 compared with untreated condition unless otherwise indicated. Data were analyzed using a one-way ANOVA with Tukey's multiple comparison test. Negative control treatment is made of serum free media and contains no addition of α-synuclein. 2.5 μM of monomeric α-synuclein were added in the Monomers condition and in the Monomers/PFF with the addition of 500 nm of pre-formed fibrils, while in the PFF condition only 500 nm of pre-formed fibrils was added to the culture media.

We hypothesized that sustained addition of exogenous α-synuclein to organotypic slices would induce abnormalities comparable to those previously observed in cellular and animal models as well as patients. First, we determined the appropriate protein concentrations using ranges previously reported for *in vitro* studies as a guide (35–37). We then treated slices with monomers (2.5 μM), sonicated pre-formed filaments (PFF, 500 nM) or a combination of both a starting at day 14 DIV (57 total DIV) then assessed whether α-synuclein treatment induced the molecular changes and characteristic pathology associated with PD. Thus, we performed immunohistochemistry using specific antibodies targeting phosphorylated α-synuclein at serine 129 (pS129), the hallmark pathological marker of PD. Consistent with previous results, we observed the appearance of round and rod-like α-synuclein structures that resemble in part, the morphology of LB and LN pathology observed in PD cases (Figures 2A,B). As expected, we observed no α-synuclein accumulation in tissue sections cultured with monomeric α-synuclein alone or untreated negative controls during the time period tested (Figure 2C). To better visualize α-synuclein inclusions at high resolution, we performed transmission electron microscopy (TEM) and observed the presence of large dense spherical aggregates within the neuronal soma. Notably, these inclusions were often found within double membrane vesicles, likely corresponding to autophagosomes (Figures 2D–F). Consistent with the results above, TEM did not reveal any protein aggregates in slices treated with monomeric α-synuclein or untreated negative control conditions (data not shown).

We next quantified exogenous α-synuclein assemblies within the tissue using a semi-quantitative immunofluorescence analysis of total α-synuclein proteins. We observed a significant increase in α-synuclein accumulation within the tissue that was highest for PFF treated slices compared to the monomeric/PFF combination (*p* < 0.01) or monomeric treatments (*p* < 0.001) relative to untreated controls (*p* < 0.0001; Figure 2G; Supplementary Figures 1A–D). Correspondingly, we observed higher pS129 fluorescence in tissue sections treated with PFF alone (*p* < 0.01) compared to the monomer/PFF combination (*p* < 0.0001; Figure 2H; Supplementary Figures 2A–D). Moreover, consistent with the increase in pS129 levels in both PFF treated conditions within the tissue (PFF, monomers/PFF), we observed a significant increase in the levels of protein aggregates within the supernatant (*p* < 0.0001) as measured with the fluorometric ProteoStat® assays (Figure 2I). As expected, this was evidently higher than monomeric α-synuclein treatments (Figure 2I). Next, we quantified the presence of α-synuclein secreted into the extracellular medium using Western blot analysis. Interestingly, we observed similar levels of total α-synuclein within the extracellular medium in both PFF treated groups (PFF or monomer/PFF) but these levels were significantly greater than monomer treated or untreated negative control slices (*p* < 0.0001; Figure 2J). Analysis of pS129 immunoreactivity in supernatants also showed significant increase after treatment with PFF alone (*p* < 0.001) and even higher with the monomer/PFF combination (*p* < 0.0001) (Figure 2K). As expected, no significant pS129 immunoreactivity was observed in monomer treated conditions or negative untreated controls (2K). Taken together, our results demonstrate that exogenous human α-synuclein is actively undergoing aggregation and post-translational modifications within this model. From this point onward, we will refer to the model presented and characterized here as the ExoSynOSlices (ESOS) model.

### Increased Cellular Death Observed Within the ESOS Model Following α-Synuclein Treatment

**Figure 3:**
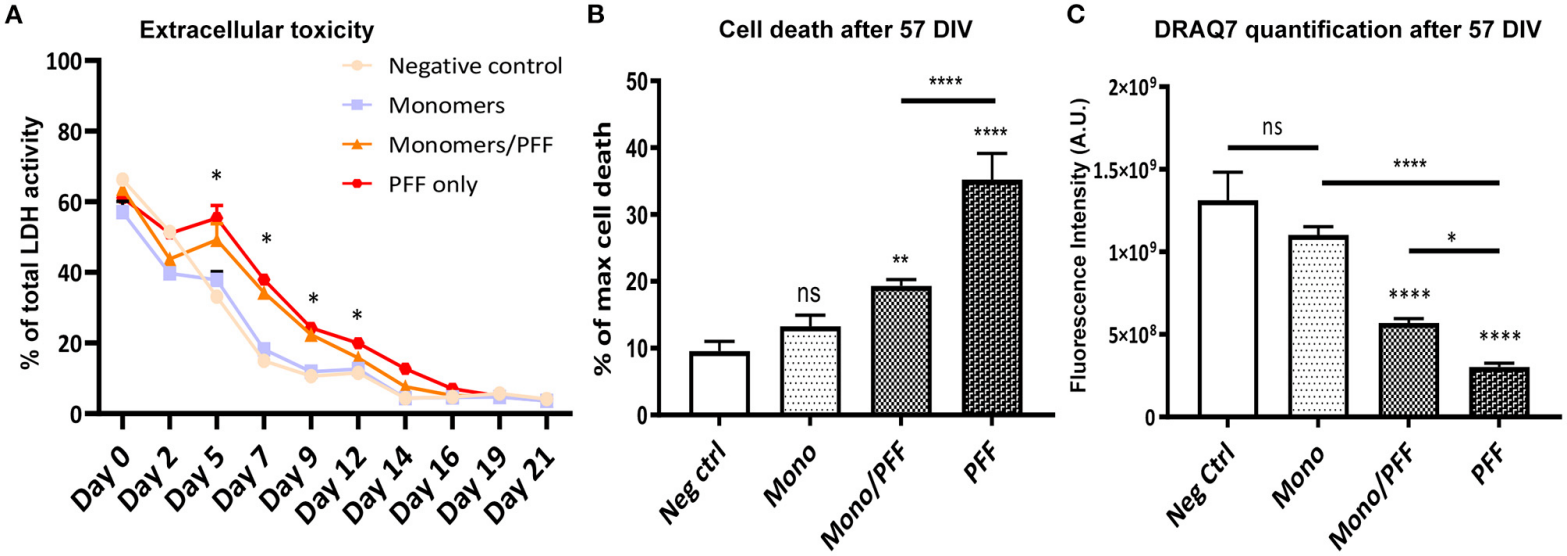
α-synuclein treatment promotes toxicity and cellular death. **(A)** Lactate dehydrogenase (LDH) activity in culture medium from organotypic hippocampal slice cultures (Light blue/squares-Negative control; Gray/circles-Monomers only; Orange/triangles-Monomers/PFF; Red/hexagons-PFF only), (*n* ≥ 3). Significance levels are for Monomers/PFF and PFF conditions compared to negative control and Monomers only condition. **(B)** Propidium iodide cell death quantification for each treatment group at 57 DIV (*n* ≥ 11). **(C)** DRAQ7 quantification of total number of cells for each treatment group at 57 DIV (*n* ≥ 7). Values in bar charts and LDH activity time course represent mean ± S.E.M. of at least three independent experiments. *p < 0.05, **p < 0.01, ****p < 0.0001 compared with untreated condition unless otherwise indicated, data were analyzed using a one-way ANOVA with Tukey's multiple comparisons test. Values in the line chart represent mean of at least three independent experiments data were analyzed using a mix-model two-way ANOVA with Bonferroni's multiple comparisons test.

Given the increase in phosphorylation of α-synuclein (pS129) within the tissue and extracellular milieu, we next investigated whether this pathological phenomenon correlated with cellular toxicity, structural abnormalities and/or increased cellular death. To first explore the effects of α-synuclein treatment on cellular toxicity, we measured the levels of cytosolic LDH released by organotypic slices in all experimental conditions. We observed significant changes in extracellular LDH concentration in the monomer/PFF combination and PFF conditions starting at 5 days post α-synuclein treatment compared to monomeric conditions (*p* < 0.01, Figure 3A). However, no difference was observed after 14 days post α-synuclein treatment (Figure 3A). We then measured propidium iodide (PI) uptake to determine the extent of overall cell death associated with α-synuclein exposure at the end of the experimental period (57 DIV). Propidium Iodide is a DNA stain which allows to differentiate healthy and death cells based on membrane integrity. In line with our previous observations, we observed an increase in cell death in slices challenged with monomer/PFF (*p* < 0.01) and significantly higher levels in PFF treated conditions (*p* < 0.0001; Figure 3B; Supplementary Figures 3A–D). To determine if cell death correlated with higher toxicity levels, we quantified the total number of cells remaining at the end of the experimental period (57 DIV). Consistent with levels of α-synuclein phosphorylation and increased cellular toxicity, we recorded a significant decrease in the total number of cells treated with both monomers/PFF and significantly higher for PFF alone (*p* < 0.0001; Figure 3C). As expected, no significant differences were observed in numbers of cells between tissues treated with monomers and untreated negative control slices (Figure 3C).

### Increased Autophagy Is Observed in Organotypic Slices Treated With α-Synuclein Assemblies

**Figure 4:**
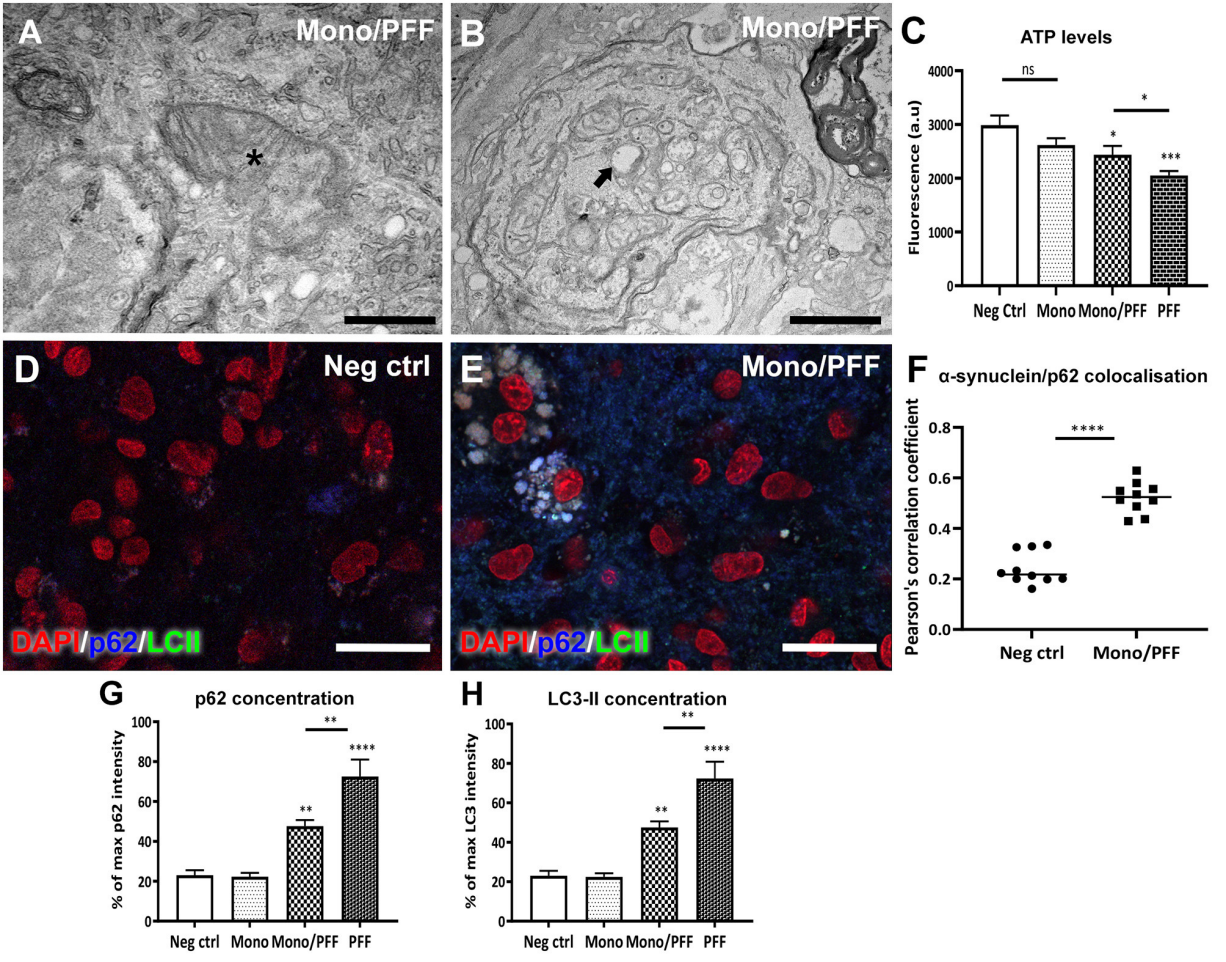
Treatment with monomeric/PFF α-synuclein induce mitochondrial toxicity. **(A)** Representative TEM images showing structural mitochondrial damage (asterisk) in organotypic slice cultures induced by treatment with monomeric/PFF α-synuclein treatment after 57 DIV. Scale bar = A 0.5 μm **(B)** Double membrane vacuoles containing electron dense material (arrow) and compressed or swollen mitochondria with severe loss of visible cristae and an empty matrix in with visibly distorted and swollen cristae. Scale bar = 1 μm **(C)** Aggregated a-synuclein decreases ATP levels, the reduction in ATP detected indicate that primary necrosis is taking place. Quantification of ATP detected using the Mitochondrial ToxGlo™ Assay at 57 DIV, (*n* ≥ 5). **(D)** Confocal immunofluorescence analysis of untreated negative control tissue or **(E)** treated with monomers/PFF showing the co-localization of p62 (blue) and LC3-II (green) and counterstained with DAPI (red). Scale bar = 30 μm. **(F)** Co-localization between α-synuclein and p62 using confocal microscopy (63 × ) were analyzed with Pearson correlation coefficient (P) after deconvolution using Huygens of 10 representative images for each condition **(G)** Average fluorescent intensity of p62 in organotypic slice cultures with different α-synuclein treatments (*n* ≥ 6). **(H)** Average fluorescent intensity of LC3-II fluorescence in organotypic slice cultures with different α-synuclein treatments (*n* ≥ 6). Data were analyzed using a one-way ANOVA with Tukey's multiple comparisons test. Values in bar charts and scatterplots represent ± S.E.M. of at least three independent experiments. *p < 0.05, **p < 0.01, ***p < 0.001, ****p < 0.0001 compared with untreated condition unless otherwise indicated.

Next, we explored potential changes in tissue ultrastructure using TEM imaging. Our analysis of tissue sections treated with monomer/PFF combination or PFF alone confirmed an overall increase in structural abnormalities affecting axons and mitochondria (Figures 4A,B). We observed numerous double membrane vacuoles containing electron dense material (arrow, Figure 4B) and compressed mitochondria with visible loss of cristae (asterisk, Figure 4A) indicating that exogenous α-synuclein accumulation likely affects the autophagic degradation system. Disturbance in mitochondrial homeostasis and lysosomal dysfunction have also been described in PD pathogenesis (25, 38). Thus, we next investigated the mitochondrial morphology and viability within the ESOS model by measuring the respiratory ATP activity levels using the mitochondrial ToxGlo™ Assay. Consistent with the results above, mitochondrial ATP levels detected in slices treated with monomers or untreated controls were substantially higher compared to slices treated with the combination of monomer/PFF (*p* < 0.05) or PFF alone (*p* < 0.001) at DIV 57. Notably, ATP levels in PFF treated slices were significantly lower than monomers or the monomers/PFF combination (Figure 4C). These results suggest that aggregated α-synuclein (PFF) is necessary and sufficient to modulate mitochondrial dysfunction.

We then investigated the endogenous substrate of autophagic degradation marker p62 and the lapidated form of LC3 (LC3-II) found in autophagosome membranes using immunofluorescence analysis. We observed an overall diffuse staining between p62 and LC3-II in monomer treated slices as well as untreated negative controls (Figure 4D), whereas the monomeric/PFF or PFF treatments revealed a clear overlap between p62 and LC3-II (Figure 4E). We then analyzed the co-localization between p62 with α-synuclein as evidence for autophagy activity (39). Thus, we obtained a Pearson correlation coefficient (P) after deconvolution using Huygens of 10 representative images for each condition showing higher co-localization in monomer/PFF combination compared to the negative controls (*p* < 0.0001, Figure 4F). Moreover, immunofluorescence analysis of tissue sections treated with monomer/PFF (*p* < 0.01), or PFF (*p* < 0.0001) further revealed a significant increase in p62 levels while no significant differences were observed between monomers or untreated negative controls (Figure 4G). Consistent with the levels of p62, we also detected a significant increase in the LC3-II concentration upon administration of monomers/PFF or PFF alone (*p* < 0.01 and *p* < 0.0001; Figure 4H). Taken together, the ESOS model described here demonstrates that organotypic slice cultures can mimic in part, the pathological mechanisms observed in cellular and animal models of PD as well as in human PD brains.

## Discussion

The relationship between the progressive deposition of α-synuclein, motor dysfunction and cognitive decline have been highlighted for multiple α-synucleinopathies including PD and dementia with Lewy bodies (1–3). α-synuclein is abundantly present in the CNS where it is a critical mediator of synaptic vesicle trafficking (40). Although the mechanisms related to the pathology and the spreading of misfolded α-synuclein have been primarily investigated within the basal ganglia due to the predominance of dopaminergic neurons with this structure (38, 41, 42), the hippocampal region is increasingly recognized as one of the structures also affected by early cellular degeneration in synucleinopathies (43). Indeed, α-synuclein pathology has been described within CA1 and CA3 regions of the hippocampus, with recent data suggesting that decreased activity within these regions correlates with α-synuclein accumulation (43–46). These studies emphasize an accumulating interest for hippocampal based platforms to study synuclein pathology accumulation in the brain.

In the present study, we characterized an interface method of culturing organotypic hippocampal slices to model α-synuclein accumulation for extended periods of time. Our anatomical and biochemical analysis confirmed that our newly established protocol is fitting for long incubation periods (up to 57 DIV) with hippocampal slices maintaining well-preserved physical architecture, normal electrophysiological responses and importantly, limited cellular death in control conditions. Thus, this model provides a good system to study the dynamics of α-synuclein pathology during experimental manipulation, making it possible for long-term studies starting even before the point where accumulation causes toxicity. In organotypic slices, aggregated α-synuclein (PFF) or a combination of monomers/PFF were actively taken up by neuronal cells, leading to the formation of round or rod-like inclusions that were positive for phosphorylation at serine 129 (pS129). Pathological structures that resemble in part, the hallmark LB and LN inclusions observed in the brains of postmortem PD patients (1–3, 7, 47–50). Moreover, our biochemical analysis further demonstrated that although similar levels of aggregated protein were observed within the tissue between different PFF treatments (PFF and monomeric/PFF), PFF treatment alone significantly decreased ATP production, increased cellular toxicity and cellular death compared to the monomer/PFF assembly combination. Interestingly, the lower levels of toxicity observed between the monomer/PFF combination correlated significantly with decreased levels of pS129 immunoreactivity within the tissue but higher within the supernatant. These results suggest that modified α-synuclein species (pS129) within the tissue may play a critical role in cellular toxicity. It is also possible that the increased levels of total α-synuclein aggregates observed within the PFF treated tissue also play a role in this process. These observations are likely corroborated by the lower cellular toxicity observed following monomeric/PFF treatment leading to an increase in pS129 immunoreactivity within the extracellular milieu but lower levels within the tissue, suggesting that successful clearance of pS129 α-synuclein species may prevent cellular toxicity.

As expected, treatment with monomers did not induce any toxicity or cell death compared to untreated negative control conditions, a result in line with previous *in vitro* (22, 35, 37) and *in vivo* (25, 38) observations. It is worth noting that while optimizing this model system, we detected cellular toxicity with lower α-synuclein concentrations for monomers/PFF combination and PFF conditions alone, but in the absence of LB-like pathology (data not shown). This observation would suggest that cellular toxicity induced by α-synuclein accumulation is not primarily driven by LB pathology but rather by an ongoing formation of protein aggregates as suggested by studies on acute effects of aggregated α-synuclein (36). In addition, our results show that the long-term, repeated addition of monomer/PFF by yet unknown mechanisms, cause increased extracellular α-synuclein in conditioned media. This suggests that the different aggregate combinations interact differently with innate mechanisms such as seeding, resulting in α-synuclein conformations that are likely more difficult to take up or easier to secrete.

Given the highly aggregated forms of fibrillar α-synuclein assemblies cultivated with hippocampal tissue, limited protein clearance compared to monomeric α-synuclein proteins was observed. This led to an increase in p62 and LC3-II proteins, indicating a reduced cellular recycling capacity to remove aggregated structures. Indeed, the increased levels of p62 and LC3-II indicate decreased activation of the autophagy pathways (51) and is comparable to reports from hippocampal neuronal cells (49) and autophagy-deficient mice (52). Moreover, we also detected direct co-localization between α-synuclein inclusions and p62 proteins indicating impaired autophagic turnover, further contributing to an increase in cellular toxicity and cell death as previously described (41, 49, 50, 53). Interestingly, we also noted an inverse correlation between co-localization of p62 and LC3-II and the presence of inclusion bodies within the tissue, corroborating the hypothesis that changes in autophagy activation may play a role in the generation of LB formation as previously reported (54–58). Finally, we observed reduced ATP levels that were highest for PFF treated slices, a result in line with the increased toxicity observed compared to monomers or the monomer/PFF combination. Moreover, our ultrastructural analysis of tissue sections revealed in some instances, multiple mitochondria engulfed in autophagosomes, suggesting disturbances of the autophagic degradation system. Our findings are in line with results from *in vitro* and animal models of PD where mitochondrial pathology has been reported as the disease progresses (58–61). However, further characterization is required to elucidate the relationship between α-synuclein accumulation and mitochondrial damage within this model under these pathological conditions. Nonetheless, using organotypic hippocampal tissue slices incubated for extended time periods, we demonstrate that pathological α-synuclein aggregation modulates disruption in mitochondrial dynamics and impairs the autophagy-lysosomal pathway.

Numerous research groups have successfully used organotypic slice cultures in experimental settings including biochemical studies, calcium imaging, live imaging, or CNS diseases (11, 13, 14). In PD, several groups have focused on α-synuclein seeding and aggregation (24, 25) and provided useful information as to seeding properties of different α-synuclein species in the CNS, in the days after α-synuclein injections. This timeframe was extended to 3–5 weeks by a recent study by Barth et al. (26), demonstrating the seeding properties of α-synuclein following injection in murine and human brain slices. In line with the results presented herein, they show the accumulation of phosphorylated (pS129) α-synuclein as well as neurodegeneration, in their case indicated by increased release of neurofilament light chain protein (62). Overall, the ESOS model presented herein provides the possibility to study the cumulative effect of α-synuclein over extended periods of time for up to 8 weeks (57 DIV) with α-synuclein treatment added at every media change. This method avoids tissue damage that may potentially be induced by injections. Thus, the method developed herein makes it possible to investigate the effect of exogenous α-synuclein in concentrations that do not cause acute toxicity allowing to assess the impact on cell viability over long time periods, allowing studies of the cumulative effects of α-synuclein in a complex environment. As α-synuclein is naturally present in the brain, it is not its presence *per se* that causes cell death, but the gradual aggregation, whether this is due to a lack of degradation, seeding or alternative explanations currently being investigated (63–65). This model demonstrates a progressive accumulation of PD-like associated inclusions and recapitulates key molecular and structural features of PD pathology. Thus, this model allows studies of pathological events starting at the point where accumulation causes toxicity or even earlier and is suitable for early screening of compounds that may interfere with such molecular processes. In addition, the ESOS model offers a reliable replacement option for *in vivo* experiments which do not include behavioral studies, thus allowing to reduce the numbers of experimental animals needed in compliance with the 3R principles (66, 67).

## Data Availability Statement

The original contributions presented in the study are included in the article/Supplementary Material, further inquiries can be directed to the corresponding author/s.

## Ethics Statement

The animal study was reviewed and approved by Linköping regional ethics committee for animal research.

## Author Contributions

SM designed the project and experiments and performed the analysis. MH supervised the project. UK and SM isolated the organotypic slices with SM and FR culturing tissue sections. SM and FR carried out the experiments. UK performed the electrophysiological recordings. SM, FR, NA, and JR contributed to sample preparation. SM, FR, NA, UK, JR, and MH interpreted the results. SM, NA, JR, and MH drafted the manuscript. All authors provided critical feedback and edited the final version of the manuscript.

## Funding

Funding was generously provided by the Swedish Research Council, The Swedish Brain foundation, The Swedish Parkinson's Foundation, The Östergötland Research Foundation for Parkinson's Disease, The Swedish Alzheimer's Foundation, the Swedish Dementia Foundation, the Hans-Gabriel and Alice Trolle-Wachtmeister Foundation for Medical Research, Linköping University and Region Östergötland. The funding agencies were not involved in the design or interpretation of the study.

## Conflict of Interest

The authors declare that the research was conducted in the absence of any commercial or financial relationships that could be construed as a potential conflict of interest.

## Publisher's Note

All claims expressed in this article are solely those of the authors and do not necessarily represent those of their affiliated organizations, or those of the publisher, the editors and the reviewers. Any product that may be evaluated in this article, or claim that may be made by its manufacturer, is not guaranteed or endorsed by the publisher.
