# Supplementary material for: Exposure of α-Synuclein Aggregates to Organotypic Slice Cultures Recapitulates Key Molecular Features of Parkinson's Disease
Source: Front Neurol. 2022 Feb 16;13:826102. doi: 10.3389/fneur.2022.826102 (PMC8925863; doi:10.3389/fneur.2022.826102)
Supplement: Supplementary file 1 [file presentation_1.pdf]

Exposure of α-synuclein aggregates to organotypic slice cultures recapitulates the molecular features of Parkinson's disease

Serge Moudio[1*], Fredrik Rodin[1], Nazira Jamal Albargothy[1], Urban Karlsson[2], Juan F. Reyes[1], Martin Hallbeck[1*]

[1]Department of Clinical Pathology and Department of Biomedical and Clinical Sciences, Linköping University, Linköping, Sweden

[2] Department of Biomedical and Clinical Sciences, Linköping University, Linköping, Sweden

*** Correspondence:**
Martin Hallbeck
martin.hallbeck@liu.se

Serge Moudio
serge.moudio@uclouvain.be

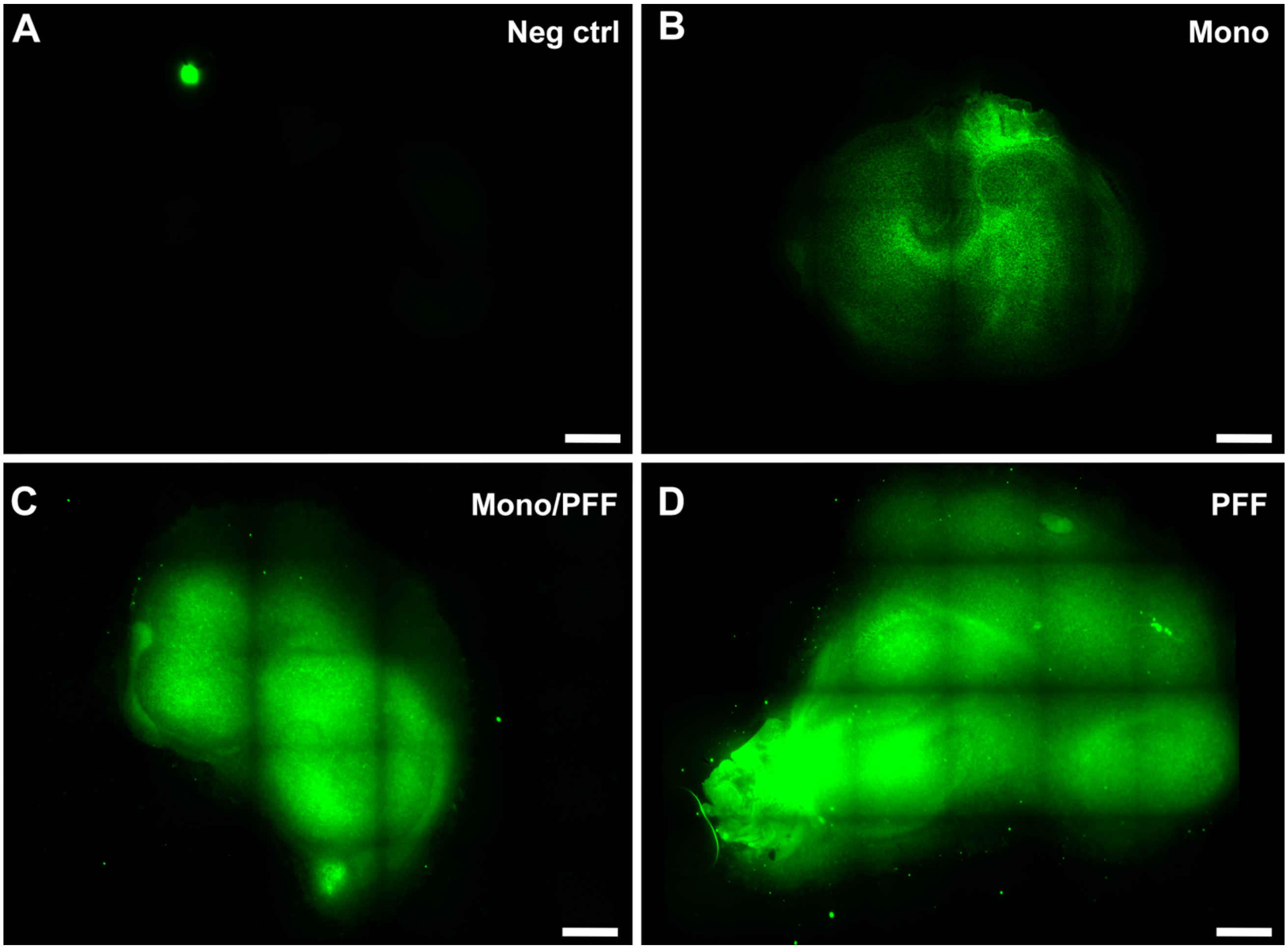

Supplemental Figure 1. α-synuclein accumulation within organotypic hippocampal slices cultured for 57 DIV. Representative (10x) immunofluorescent images after staining for α-synuclein in control slice (A) or treated with monomers (B) monomers/PFF (C) or PFF (D) respectively. Scale bar = 500 µm.

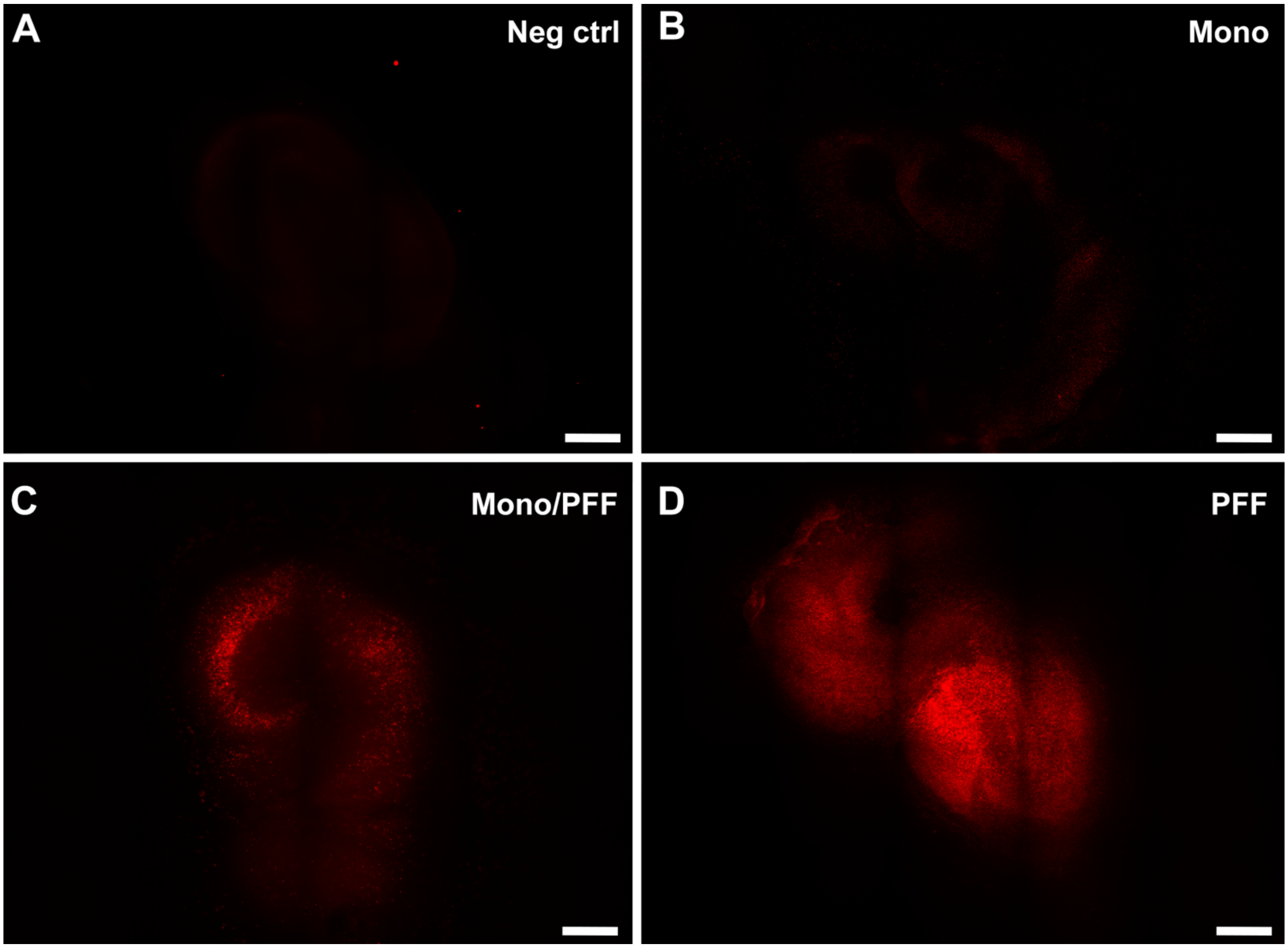

Supplemental Figure 2. accumulation of phosphorylated α-synuclein within organotypic hippocampal slices cultured for 57 DIV. Representative (10x) immunofluorescent images after staining for pS129 α-synuclein in control slice (A) or treated with monomers (B) monomers/PFF (C) or PFF (D) respectively. Scale bar = 500 µm.

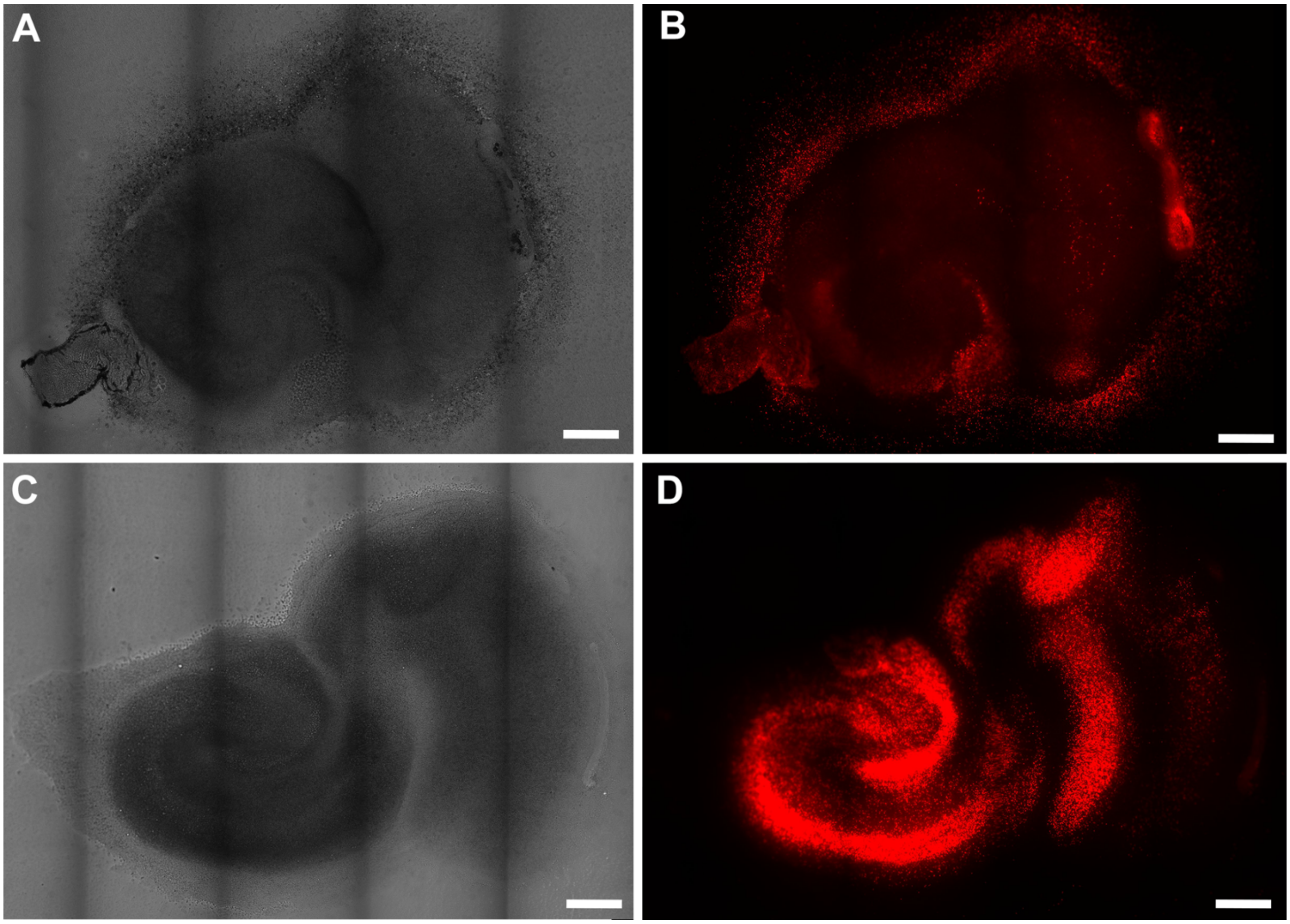

Supplemental Figure 3. Mono/PFF induces cell death in organotypic slices cultured for 57 DIV. Representative (10x) bright field (A and C) and fluorescent propidium iodide (B and D) images of organotypic hippocampal slices for negative control (A and B) and Mono/PFF (C and D) after 57 DIV. Scale bar = 500 μm.
